# Survey on the management of acute first-time anterior shoulder dislocation amongst Dutch public hospitals

**DOI:** 10.1007/s00402-015-2156-3

**Published:** 2015-02-21

**Authors:** T. D. Berendes, P. Pilot, J. Nagels, A. J. H. Vochteloo, R. G. H. H. Nelissen

**Affiliations:** 1 Department of Orthopaedics, Meander Medical Centre, Maatweg 3, Postbox 1502, 3800 BM Amersfoort, The Netherlands; 2 Department of Orthopaedics, Reinier de Graaf Hospital, Delft, The Netherlands; 3 Department of Orthopaedics, Leiden University Medical Centre, Leiden, The Netherlands; 4 Orthopaedic Centre OCON, Hengelo, The Netherlands

**Keywords:** Shoulder, Dislocation, Survey, Guideline, Implementation, Treatment

## Abstract

**Introduction:**

The primary aim of this study was to record how orthopaedic surgeons are currently managing acute first-time anterior shoulder dislocation (AFASD) 8 years after introduction of the Dutch national guideline: “acute primary shoulder dislocation, diagnostics and treatment” in 2005. The second aim was to evaluate how these surgeons treat recurrent instability after AFASD.

**Materials and methods:**

An online questionnaire regarding the management of AFASD and recurrent shoulder instability was held amongst orthopaedic surgeons of all 98 Dutch hospitals.

**Results:**

The overall response rate was 60 %. Of the respondents, 75 % had a local protocol for managing AFASD, of which 28 % had made changes in their treatment protocol after the introduction of the national guideline. The current survey showed wide variety in the overall treatment policies for AFASD. Twenty-seven percent of the orthopaedic surgeons were currently unaware of the national guideline. The variability in treatment for AFASD was present throughout the whole treatment from which policy at the emergency department; when to operate for recurrent instability; type of surgical technique for stabilization and type of fixation of the labrum. As for the treatment of recurrent instability, the same variability was seen: 36 % of the surgeons perform only arthroscopic procedures, 7 % only open and 57 % perform both open and arthroscopic procedures.

**Conclusions:**

Despite the introduction of the national guideline for the initial management of AFASD in 2005, still great variety among orthopaedic surgeons in the Netherlands was present. As for the surgical stabilization technique, the vast majority of the respondents are performing an arthroscopic shoulder stabilization procedure at the expense of the more traditional open procedure as a first treatment option for post-traumatic shoulder instability.

**Electronic supplementary material:**

The online version of this article (doi:10.1007/s00402-015-2156-3) contains supplementary material, which is available to authorized users.

## Introduction

Acute first-time anterior shoulder dislocation (AFASD) is an injury that is frequently seen on the Emergency Department (ED). Shoulder dislocations comprise approximately 10 % of all shoulder trauma and approximately 50 % of all joint dislocations [1]. Reported incidence rates of shoulder dislocation vary from 8 to 48 per 100,000 inhabitants per year [2–6].

**Fig. 1:**
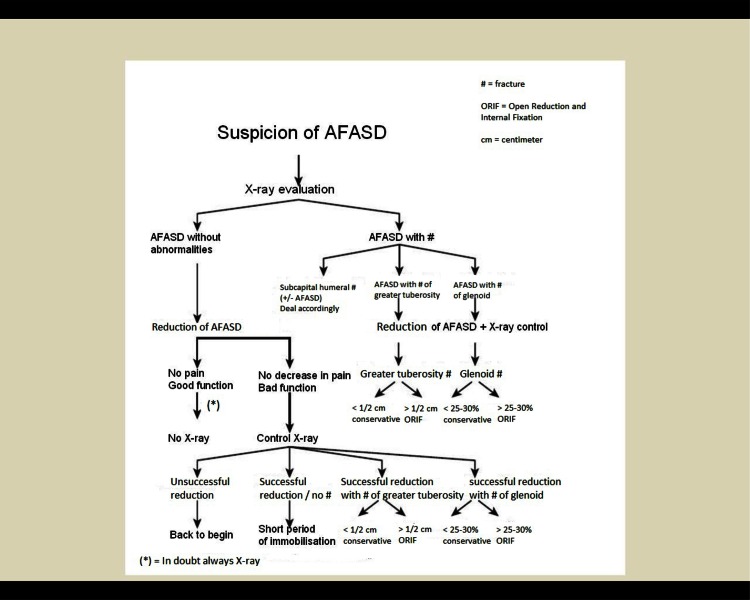
Flowchart treatment of acute primary anterior shoulder dislocation according to Dutch national guideline 2005

Previous studies showed a great variety of treatment options in managing AFASD [7, 8]. A prior Dutch questionnaire demonstrated that there was no protocol for the management of AFASD in 35 % of all consulted hospitals [1]. Therefore, it was proposed to develop a national evidence-based guideline for the management of AFASD. In 2005, the national guideline: “acute primary shoulder dislocation, diagnostics and treatment” was introduced, written by a Work Group commissioned by the Dutch Orthopaedic Association (NOV [9]). The flowchart of treatment of AFASD from this guideline is depicted in Fig. 1.

The guideline was at the request of this Working Group, assessed by a number of experts in the field. In the comment phase, the guideline was offered online to all members of the participating Dutch associations (General Surgery and its subdivision of Traumatology, General Practitioners, Physiotherapy, Radiology and Sports Medicine) and to the Work Group Shoulder and Elbow within the Dutch Orthopaedic Association itself. Hereafter, the guideline was accepted in the general assembly of the Dutch Orthopaedic Association, followed by the publication in 2005 [9].

The primary aim of this study was to evaluate how orthopaedic surgeons are currently managing AFASD, 8 years after introduction of the guideline [9]. The second aim was to evaluate how these surgeons treat recurrent instability after AFASD.

Our hypothesis was that the impact of the new guideline was small and that we would not see a uniform treatment of AFASD despite the implementation of the guideline in 2005 in the Netherlands.

## Materials and methods

An online questionnaire regarding the management of AFASD and recurrent shoulder instability was held amongst orthopaedic surgeons of all 98 Dutch hospitals (eight university hospitals, 21 teaching hospitals, 69 general hospitals). Orthopaedic healthcare is joint oriented within orthopaedic groups in hospitals, thus 1–2 surgeons perform shoulder surgery, knee surgery, etc. Orthopaedic groups in the Netherlands are mandatory to have at least three or four consultants to guaranty quality and continuity. So, the reactions given in this study are per orthopaedic group.

The Questionnaire was based on the Dutch guideline on AFASD and was made by a panel of shoulder surgeons. It consisted of 27 multiple-choice questions and one open question.

The questionnaire (in Dutch) is available online (and an English translation is given in the appendix) (http://spreadsheets.google.com/viewform?formkey=dElONW5BWEFnaEpFcWtDUDFCTzVtTWc6MA). Furthermore, two case vignettes were used as for what treatment policy the orthopaedic surgeon would do in two distinct cases of AFASD (Ron te Slaa [10]). These case vignettes were: a 17-year-old man, active high-level handball player, with persistent instability after an AFASD (after the initial dislocation, three or more dislocations or subluxations occurred) and a 47-year-old low-demanding housewife with the same clinical presentation.

## Results

Fifty-nine (60 %) orthopaedic groups of 98 orthopaedic groups (i.e. Dutch hospitals) completed the online questionnaire. As for the type of orthopaedic groups who responded, seven out of eight orthopaedic groups (88 %) from university hospitals, 14 out of 21 (67 %) orthopaedic groups from teaching hospitals and 38 of the 69 (55 %) orthopaedic groups from general hospitals responded. No private clinics responded, since they do not participate in the management of the acute shoulder dislocation in the Netherlands. Fifty-eight of the 59 responding orthopaedic groups (98 %) performed surgery for post-traumatic recurrent instability.

Table 1 shows the answers on the items of the questionnaire regarding the 2005 guideline, timing of radiographs and the treatment of AFASD. The most important findings were that 16 of the 59 (27 %) orthopaedic groups are currently unaware of presence of a national guideline and that 44 (75 %) had a local protocol for AFASD. Of these 44, 17 had adjusted their protocol after release of the national guideline in 2005. The majority of the orthopaedic groups (51/59, 86 %) make radiographs of the shoulder both pre- and post-reduction and have a standardized treatment protocol after reduction (53/59). Visible from the responses given in this study, a great variation was present in the different hospital protocols for management of AFASD.

**Table 1:** Items of the questionnaire regarding the guideline of 2005, X-rays and treatment of AFASD

Questions	Yes	No	Unknown
Guideline			
Awareness of guideline for AFASD of 2005	43	13	3
Presence of protocol for AFASD in your hospital?	44	11	4
Adjustments of local protocol after release of guideline in 2005	17	17	25
X-rays			
PRE-reduction X-rays on ED	51	7	1
POST-reduction X-rays on ED	51	5	3
Treatment			
Subsequent standard treatment after AFASD	53	5	1
Immobilization of shoulder post-reduction?	52	6	1
Physiotherapy after AFASD?	32	25	2

*n* = 59 orthopaedic groups

*AFASD* acute first-time anterior shoulder dislocation, *ED* emergency department

The anaesthetic technique for reduction of the dislocated shoulder showed large variability as well: the majority (46 %) of the respondents use a combination of different anaesthetic techniques at time of reduction, 20 % only intravenous diazepam and 10 % routinely used the intra-articular injection of lidocaine (IAL). In 4 % fentanyl and/or midazolam is used; the solitary use of any combination of paracetamol, non-steroidal anti-inflammatory drugs (NSAIDs) or morphine is used in 2 % and finally, general anaesthesia is used by 2 %. In 8 % no form of anaesthesia was used.

*The reduction technique showed also variability* Forty-seven percent of the responders answered that they used some form of combination of the four classic reposition techniques. In 17 % the Hippocrates technique was used, in 14 % the Kocher technique, 12 % the Stimson technique and 5 % the Milch technique [11]. Five percent replied that they would use another technique besides the mentioned ones in the questionnaire to reduce a shoulder dislocation in the Emergency Department, but no further specification was done.

*Immobilization technique and time showed less variability* The shoulder was immobilized for a maximum of 2 weeks by fifty-nine percent of the orthopaedic groups. The remaining 41 % advises immobilization for 2–6 weeks. The majority (97 %) immobilizes the shoulder in internal rotation, only three percent immobilizes the shoulder in external rotation.

*Follow-up and aftercare after AFASD again were very variable within the groups* Fifty-four percent of our responders claimed to perform some form of subsequent treatment after AFASD, mostly physiotherapy.

Eighty-eight percent of the respondents claimed to routinely check patients at the outpatient clinic after AFASD; 46 % within 1 week; 41 % within 2 weeks and 14 % after 6 weeks. Thirty-two surgeons (60 %) routinely refer patients to a physiotherapist after AFASD.

*Diagnostic investigation, timing and techniques for recurrent instability showed also variability* Thirty-nine percent (23/59) of the responding orthopaedic groups first refer the patient to a physiotherapist when symptoms of recurrent instability occur; additional diagnostic evaluation before further (conservative) treatment was started by 36 orthopaedic groups (66 %). A MRI scan is the favourite diagnostic tool (98 %, 58/59), in the majority with intra-articular contrast (55/58).

*The surgical treatment options for recurrent instability after AFASD showed remarkable variations* In four orthopaedic groups (7 %), only an open stabilizing technique was used; 93 % (54/58) used an arthroscopic technique as a primary treatment for recurrent instability. In the latter group, 61 % (33/54) performs open stabilizing techniques as well, sometimes for the primary cases.

The modified open Bankart repair is performed in the majority (54 %) of cases, 16 % of the surgeons prefer a Putti-Platt procedure; 14 % a Bristow–Latarjet procedure; 14 % a T-shaped capsular shift and 2 % a Weber osteotomy.

For refixation of the labrum (either open or arthroscopically), 40 % (23/57) of the respondents uses non-absorbable suture anchors as fixation technique, 47 % (27/57) uses absorbable suture anchors and 13 % (7/57) uses capsulolabral sutures (without an anchor).

Forty-five percent (26/58) of all surgeons use a standard postoperative follow-up of 6 months, fifty percent (29/58) 1 year and five percent of the surgeons (3/58) have a follow-up of more than 1 year.

*Looking at the factors of influence on decision-making, logical differences were given* Eighty-eight (52/59) percent of the responders indicated that age was an important factor in decision-making for further treatment. Level of sport activity plays an important role in the treatment process in 86 % (51/59) of the respondents, 84 % makes a further differentiation in contact versus non-contact sports and throwing versus non-throwing.

On the question: “how many dislocations must a patient have been through to decide to intervene surgically?” two percent (1/50) of the respondents replied one dislocation; 34 % (17/50) two or more, 40 % (20/50) three or more and 24 % (12/50) over four dislocations.

Spontaneous dislocation at rest or while sleeping is a reason to perform surgery for 83 % (49/59) of the respondents.

As for the case vignette of the 17-year-old man, fifty-one out of 59 respondents (86 %) answered to perform a stabilizing procedure, of which 47 % (28/59) would perform an arthroscopic procedure, 8 % (5/59) would perform an open procedure and 31 % (18/59) would perform another type of stabilizing procedure. The remaining eight respondents (14 %) preferred a conservative treatment.

As for the second case vignette, thirty out of 57 respondents (53 %) would perform a stabilizing procedure, of which 33 % (19/57) would perform this arthroscopically, 16 % (9/57) would perform a non-defined type of stabilizing procedure and 4 % (2/57) would perform an open procedure. The remaining twenty-seven respondents (47 %) preferred a conservative treatment.

## Discussion

A great variety among orthopaedic surgeons in the Netherlands for the initial management of AFASD was found, despite the introduction of the national guideline in 2005.

A quarter of the Dutch orthopaedic groups are currently unaware of the presence of the national guideline implemented in 2005 and three quarters had a local protocol for AFASD in their hospital of which a minority had adjusted their protocol after release of the new guideline.

These findings are in line with our hypothesis that the impact of the new guideline would be small and would not lead a uniform treatment of AFASD.

Several reviews have shown that guidelines have only been moderately effective in changing the process of care, and that there is much room for improvement [12]. Implementation of medical guidelines poses difficulty which can be related back to several constraints [13–15]. A prominent barrier for implementation is lack of agreement with guideline recommendations. Lack of applicability is another important barrier to guideline adherence. Environmental barriers, particularly organizational constraints, are another often-perceived group of barriers to implementation. Moreover, lack of collaboration with other types of healthcare professionals and lack of motivation, time, resources and reimbursement are also shown as a barrier to implementation [12]. Carlson’s conducted review in 2007 identified six themes of barriers to the implementation of guidelines among general practitioners (GP): the content and the format of a guideline, GPs individual experience, preserving the doctor–patient relationship, professional responsibility, and practical issues [16].

So, one can imagine, with AFASD with its widespread clinical presentation of symptoms between different types and demanding patients with different types of treatment options, that a guideline for AFASD will be difficult to implement in daily practice, unless there is conclusive scientific evidence that a particular treatment is best for AFASD. And even then, it appears that the implementation of a protocol is difficult. If guidelines are made, effort has to be made on implementing them in daily practice.

A survey by te Slaa in 2003, prior to the introduction of the national AFASD guideline, demonstrated that 65 % of the reviewed Dutch hospitals had a protocol for AFASD (response rate 73 %, of 74 Dutch hospitals) [1]. These protocols were different, because they have been made individually per clinic based on their own interpretation of knowledge and understanding on dealing with AFASD at that time and place. Of course, this is accompanied by a degree of heterogeneity between the individual protocols. Our study found that currently 75 % had an AFASD protocol that was adjusted in 29 % after the introduction of the guideline. Therewith, the impact of the introduction of the guideline is small; a 10 % increase of presence of an AFASD protocol. Furthermore, large differences in management of AFASD are still present.

A similar wide variety among trauma clinicians in managing AFASD was found in surveys conducted in the UK and Germany [8, 17].

### Anaesthetic technique

The guideline stated that it should be considered to give IAL as a local analgesic and that in case of a failed first reduction, enhanced analgesia, sedation and/or anaesthesia might be used.

The UK survey (2006) showed also that 10 % of respondents used intra-articular injection of lidocaine (IAL) prior to reduction [8], comparable to our findings. The German survey (2001) does not describe the analgesic management [17].

Two randomized controlled trials demonstrated that a combination of sedation and analgesia resulted in a higher reduction rate, but with more complications (respiratory depression, nausea and vomiting) when using sedation [18, 19]. IAL is a safe and effective method that contributes to a successful and less painful repositioning promoted by Matthews, Gleeson and Suder [20–22]. It has been shown that IAL has less side effects without differences in time to reposition, difficulty of repositioning or subjective pain perception and a shorter stay on the ED compared to intravenous sedation [20, 23].

### Reduction technique

Reduction techniques can be divided into four groups as described by Riebel and McCabe [11].

The traction method (Hippocrates, Stimson), the leverage method (Kocher, Milch), scapula manipulation method and the last group is the combination of the prior three [11]. The guideline states that no reduction technique is considered to be superior and to use the technique each practitioner is known and familiar with, which is in line with the findings of our survey.

### Immobilization

If immobilized, the optimum position and duration of immobilization is still not known [24].

With regard to the duration of immobilization, Kiviluoto showed that the redislocation rate was higher in patients under 30 years compared to older patients and that in the under 30-year group the redislocation rate was higher in those that were immobilized for 1 week compared to those subjected to 3 weeks’ immobilization [25]. Itoi et al. showed a better outcome after a first-time anterior shoulder dislocation after immobilization of the shoulder in external rotation and abduction when the shoulder is immobilized for at least 3 weeks [26–28]. However, Liavaag et al. [29] refute this later on in their article in 2011.

In our survey, we found a large preference for immobilization of the shoulder in internal rotation position.

The guideline indicates that immobilization in general is not proven useful after AFASD as there is no correlation between recurrence and the length of immobilization [30, 31] and that there is no preference for the position of immobilization [9].

### Follow-up and aftercare after AFASD

The vast majority of the consulted clinics performed some kind of follow-up after AFASD, which is according to the guideline, stating that after the immobilization period, it is necessary to determine the extent of shoulder function both in an active and passive way. Patients should be able to be completely pain-free with a full active range of motion of the shoulder within 6 weeks after a shoulder dislocation. In the guideline, physiotherapy is not recommended for a patient with an uneventful course of AFASD. This is in conflict with the survey outcome. This is probably because of the expectations of most patients to receive some form of rehabilitation.

### Recurrent instability: diagnostics

In the guideline, additional imaging is recommended in case of persistent pain and/or loss of function of the shoulder approximately 2–6 weeks after AFASD. In contrast to this, we found that in daily practice, patients are referred to a physiotherapist when signs of recurrent instability occur. If additional imaging is performed, MR arthrography is the examination of choice of the large majority, in line with guideline. Only when rotator cuff pathology is suspected, ultrasound examination is the first choice. This is in line with many (more recent) studies [32–36].

### Recurrent instability after AFASD: surgical treatment

The guideline does not advise on specific surgical techniques, only on timing. A ‘wait and see’ period after AFASD before deciding to operate, even in young athletes, is advised.

This advice is because of the relatively low redislocation rate in the average patient (26 % in a normal population) [9]. However, the redislocation rate is much higher (up to 68 %) in younger physically active patients. Therewith, surgical stabilization after the first dislocation in this specific group is currently still subject of scientific debate [37–39]. In our study, arthroscopical procedures were clearly more performed than the (traditional) open stabilization procedures as surgical treatment for recurrent instability. As the results of arthroscopic repair have greatly improved, arthroscopic techniques have driven off the open techniques [40, 41]. Historically, the open procedures had a lower recurrence rate compared to the present arthroscopic stabilizing techniques [42–44]. With newer studies, however, more evidence is found for similar long-term clinical outcomes, with no significant difference in the rate of recurrent instability and or clinical outcome scores [45, 46].

Looking at the open techniques in our survey, there was a clear preference for the open (modified) Bankart technique (54 %) compared with the Bristow–Latarjet procedure (16 %) reflecting international preferences [7, 47]. Furthermore, it was interesting to see that the Putti-Platt procedure is still used quite often (16 %), more than in the German survey (8 %) [7, 17].

This procedure, however, has a high correlation rate with loss of motion (especially external rotation) and osteoarthritis on the long term [48, 49]. Also notable was the number of surgeons (12 %) using capsulolabral suture repairs which are proven inferior to (non-) absorbable suture anchors [50–52].

Our findings with regard to timing of surgical treatment after AFASD were conflicting. In the survey itself, 2 % of the respondents would perform direct surgical repair after one dislocation. However, only 14 % of all surgeons were in favour of the ‘wait and see’ treatment for the active, young patient in case vignette 1. So, age and level or types of sport activity were found to be important issues in decision-making of (surgical) treatment.

In the German study, 73 % of the surgeons would treat a young, athletic patient (<30 years old) surgically already after the first dislocation (and 98 % in case of recurrent instability). The same patient with a moderate level of sport activity would be treated conservatively in 67 % of cases (14 % in case of recurrent instability). The level of sports is therewith important in the German setting [7].

Clinical practice guidelines are commonly regarded as useful tools for quality improvement. However, the impact of this guideline on clinical practice for management of AFASD is not optimal because of the many constraints for implementation. Uniformity in the treatment of AFASD is difficult to achieve, despite evidence-based medicine, which might be due to the fact that most advice in the guideline is based on level III or IV evidence or expert opinion. Second, even if level I evidence is present, implementation is difficult.

Based on current literature, we suggest a future guideline on AFASD should propagate the use of IAL as anaesthetic technique and a short period of immobilization after AFASD. It should advise better on when (not) to use a specific surgical technique. Finally, it could be considered to treat young and competitive patients surgically more early as of their high recurrence rate.

To conclude, our survey revealed a great variety among Dutch orthopaedic surgeons with regard to the management of AFASD, despite the introduction of a national guideline in 2005.

As for the surgical stabilization technique, the vast majority of the respondents are performing an arthroscopic shoulder stabilization procedure at the expense of the more traditional open procedure as a first treatment option for post-traumatic shoulder instability.

## Electronic supplementary material

Below is the link to the electronic supplementary material.

Supplementary material 1 (DOCX 20 kb)
