# Supplementary material for: Survey on the management of acute first-time anterior shoulder dislocation amongst Dutch public hospitals
Source: Arch Orthop Trauma Surg. 2015 Feb 21;135(4):447–54. doi: 10.1007/s00402-015-2156-3 (PMC4365281; doi:10.1007/s00402-015-2156-3)
Supplement: Supplementary file 1 — Supplementary material 1 (DOCX 20 kb) [file 402_2015_2156_MOESM1_ESM.docx]

**Appendix:** questionnaire Dutch translation (online version)

<http://spreadsheets.google.com/viewform?formkey=dElONW5BWEFnaEpFcWtDUDFCTzVtTWc6MA>..

**Appendix**: questionnaire English translation

**Questionnaire on management of Acute First-time Anterior Shoulder Dislocation (AFASD) amongst Dutch orthopaedic surgeons**

The questionnaire consists of 30 questions, the last two in the form of case vignettes.

Thank you for your effort.

* Required

Enter the name of your hospital :

1 . Are you aware of the presence of the CBO guideline “shoulder dislocation” of 2005? *

• yes

• no

• I do not know

2 . Is there a protocol available in your hospital for shoulder dislocations ? *

• yes

• no

• I do not know

3 . If so, has your protocol been changed after the introduction of the CBO guideline in 2005 ?

• yes

• no

• I do not know

4 . Is an X-ray being performed routinely before reduction in the emergency department?*

• yes

• no

• I do not know

5 . Is an X-ray being performed routinely after reduction in the emergency department? *

• yes

• no

• I do not know

6. Which anesthetic technique is applied at the time of the reduction in the emergency department? *

• Lidocaine injection intra-articular in the shoulder

• Acetaminophen and / or NSAIDs and / or Morphine

• Fentanyl and / or Midazolam

• Combination of the above options

• General anesthesia

• Diazepam

• No form of anesthesia

• Other options

7 . Which repositioning technique is being performed at the emergency department? *

• method according to Hippocrates (=traction in the caudal direction in combination with slight adduction )

• method of Kocher (=external rotation, while lifting the humeral head past the glenoid rim)

• method of Stimson (=dropping arm in the prone position with approximately 10lb of weight applied to the wrist on the affected side)

• method of Milch (=elevation of the affected arm in a prone position, while the surgeon abducts and externally rotates the patient's arm into an overhead position reducing the shoulder)

• combination of the above methods

• something else

8 . Subsequent treatment of AFASD *

Is an after treatment routinely applied?

• yes

• no

• I do not know

9 . Is routinely advised to immobilize the shoulder ( post – reduction) after AFASD? *

• yes

• no

• I do not know

10 . If so, what is advised with respect to the duration of immobilisation ? *

• < 2 weeks

• 2-6 weeks

11. If so, how is the shoulder immobilised ? (Technology ) *

• internal rotation

• external rotation

12 . Is follow-up routinely being performed at your clinic after AFASD? *

• yes

• no

• I do not know

13 . If so, when? *

• 1 week post- reduction

• 2 weeks post reduction

• 6 weeks post- reduction

• > 6 weeks post- reduction

14 . Is the patient ALWAYS being referred to a physiotherapist after AFASD? *

• yes

• no

• I do not know

15 . When instability persists: *

Do you refer the patient to a physical therapist or do you first perform additional studies?

• First physiotherapy

• First additional study

16 . Additional studies: *

What type of study do you prefer after the primary X-ray investigation ?

• Ultrasound

• MRI arthrography

• MRI

• Arthroscopy

• CT scan

17 . Is surgery for shoulder instability being performed in your clinic ? *

• yes

• no

18 . If so, what kind of technique is being performed ?

• Open technique

• Arthroscopic technique

• Both techniques

19 . If open surgical stabilizing techniques are being performed, which one do you use?

• ( modified ) Bankart technique

• Putti - Platt procedure

• Bristow - Latarjet procedure

• Magnuson - Stack procedure

• Du Toit capsulorraphy

• Weber osteotomy

• T – kapsular shift

• something else

20 . Which type of labral refixation technique is being performed ?

• Unsolvable transglenoidal anchors

• Solvable transglenoidal anchors

• Capsuloligamentous labrum sutures

• Others

21 . Follow-up postoperatively:

How long is your postoperative follow-up ?

• <6 months

• 1 year

• 2 years

• > 2 years

22 . Age patient: *

Is the age of the patient important regarding your treatment process?

• yes

• no

• I do not know

23 . Age patient:

If so, on what?

• duration period physiotherapy

• duration period of immobilization

• influence on decision to operate

• more of the above answers

• something else

24 . Sport activity: *

Is the level of sport activity in the patient important regarding your treatment process?

• yes

• no

• I do not know

25 . Sport activity:

If so, do you differentiate between type of sport? (contact vs. non-contact sport )

• yes

• no

• I do not know

26 . Persistent instability *

How many shoulder dislocations before you decide on surgical intervention?

27 . Persistent instability *

Is spontaneous dislocation at rest / during sleep (after an initial traumatic dislocation) a reason for you to operate?

• yes

• no

• I do not know

28 . Persistent instability

If so, after how many spontaneous dislocations at rest do you decide to intervene ?

• 1 spontaneous luxation

• 2 spontaneous dislocations

• 3 spontaneous dislocations

• > 3 spontaneous dislocations

29 . Case vignette No. 1: *

A 17 - year-old male, active handball player with persistent instability after a traumatic anterior shoulder dislocation comes to you for consultation, subsequently proven with at least 3 or more subluxations.

What would you recommend?

30 . Case vignette No. 2: *

A 47 - year-old housewife with persistent instability after traumatic anterior shoulder dislocation comes to you for consultation , subsequently proven with at least 3 or more subluxations.

What would you recommend?
